# Exosomes from *Limosilactobacillus fermentum* Ameliorate Benzalkonium Chloride-Induced Inflammation in Conjunctival Cells

**DOI:** 10.3390/ijms252212282

**Published:** 2024-11-15

**Authors:** Kippeum Lee, Hyeonjun Gwon, Joo Yun Kim, Jae Jung Shim, Jae Hwan Lee

**Affiliations:** R&BD Center, Hy Co., Ltd., 22 Giheungdanji-ro 24 Beon-gil, Giheung-gu, Yongin-si 17086, Republic of Korea; joy4917@hanmail.net (K.L.); hjgwon@hy.co.kr (H.G.); jjshim@hy.co.kr (J.J.S.); jaehwan@hy.co.kr (J.H.L.)

**Keywords:** conjunctiva cell, exosomes, exosomes, ocular inflammation, *Limosilactobacillus fermentum* HY7302

## Abstract

Dry eye is characterized by persistent instability and decreased tear production, which are accompanied by epithelial lesions and inflammation on the surface of the eye. In our previous paper, we reported that supplementation with *Limosilactobacillus fermentum* HY7302 (HY7302) could inhibit corneal damage in a benzalkonium chloride (BAC)-induced mouse model of dry eye, through its effects in gut microbiome regulation. The aim of this study was to determine what functional extracellular substances can alter the inflammatory response of conjunctival cells. We isolated exosomes from HY7302 probiotic culture supernatant, analyzed their morphological characteristics, and found that their average size was 143.8 ± 1.1 nm, which was smaller than the exosomes from the *L. fermentum* KCTC 3112 strain. In addition, HY7302-derived exosomes significantly reduced the levels of genes encoding pro-inflammatory cytokines, including *interleukin (IL)-20*, *IL-8*, *IL-6*, and *IL-1B*, in BAC-treated human conjunctival cells. Moreover, HY7302-derived exosomes significantly increased the levels of genes encoding tight junction proteins, including *TJP1*, *TJP2*, and *occludin-1*, in Caco-2 cells. Lastly, the HY7302 exosomes reduced mRNA expression levels of *IL1B*, *IL20*, *IL6*, *IL8*, and *NFAT5* in a transwell coculture system. Our findings indicate that HY7302 exosomes have potential for use in the treatment of ocular inflammation-related dry eye disease, through gut–eye axis communication via exosomes.

## 1. Introduction

Dry eye disease (DED) is a multifactorial eye disorder characterized by symptoms of ocular dryness, dysfunctional tear production, somatosensory abnormalities, and ocular surface inflammation [1,2]. DED, also known as keratoconjunctivitis sicca, is a persistent issue that can compromise eye health and quality of life [3,4]. The worldwide prevalence of DED ranges from 5% to 50%, and the condition impacts life quality, vision, ocular function, and work productivity, as well as causing considerable pain [5,6]. DED can be caused by various environmental factors, including dry and windy conditions, air pollution, and exposure to digital screens for extended periods, which contribute to the accelerated evaporation of tears [7,8]. These environmental factors cause changes in the conjunctiva and ocular surfaces, which disrupt the tear film, leading to dryness and epithelial stress [9]. DED can be a natural part of the aging process, or attributable to various health conditions, such as chronic disease, diabetes, and long-term wearing of contact lenses or exposure to preservatives contained in artificial tear drops, such as benzalkonium chloride (BAC) [10,11]. In particular, since the effect of artificial tear drops and their side effects is only temporary, various alternative approaches to ameliorate the symptoms of dry eyes through dietary adjustment have been attempted [12]. For example, foods rich in omega-3 fatty acids, such as fish, flaxseed, and walnuts, can help improve tear production and relieve DED symptoms [13,14,15].

DED often results in an inflammatory reaction accompanied by pink eye, which is usually caused by an inflammatory response of the conjunctiva, a transparent membrane between the eyelids and the eyeball [16]. Conjunctival inflammation often leads to conjunctival edema, which significantly disrupts the ocular surface. Moreover, persistent inflammatory stimulation of conjunctival tissue can activate adaptive immune responses, resulting in chronic inflammation [9,17]. Further, it is reported that production of the inflammatory cytokines, interleukin (IL)-1, IL-6, and IL-8, in the tear membrane is increased in patients with dry eyes [18,19]. However, despite investigation of the correlation between DED and inflammation for the past 40 years, the underlying cellular and molecular level mechanisms involved have yet to be fully elucidated [9].

Extracellular vesicles (EVs) are small membrane-bound compartments released by cells, including microbes, that have important roles in intracellular and intercellular communication [20,21]. Structurally, EVs are surrounded by phospholipid bilayer membranes, and can contain proteins, lipids, DNA, mRNA, and miRNA [22,23]. Although EVs were considered “cell dust” in the past, there is now strong evidence that nanoscale EVs have pivotal roles in inter-microbial communication, as well as host-to-bacteria interactions, by mediating the diffusion of signaling molecules [24,25]. EVs are mainly distinguished by their size, and can be further delineated by their composition and function [26]. Several studies have shown that EVs are divided into the three major types of apoptotic bodies, microvesicles, and exosomes [22]. These EV subclassifications are distinguished by their size or biosynthetic characteristics, but ultimately lead to confusion in nomenclature, duplication, and confusion [27]. In particular, EVs of 30–160 nm, derived from a specific intracellular biosynthesis process, are referred to as exosomes [28,29]. The importance and biomedical effects of exosomes are established and widely described in the literature [27,30]. There is a growing appreciation of the functional importance of gut microbiota in health and disease, and the roles of microorganism-derived exosomes in tissue-to-tissue communication, horizontal gene transfer, the distribution of nutrients among communities, and inflammatory control have become the focus of considerable research attention [20,31]. In addition, exosomes secreted by non-pathogenic microorganisms, including probiotics, are reported to have various health promoting effects, for example, in skin care and aging prevention, inflammation, cardiovascular disease, and cancer [32,33,34]. Therefore, exosomes are rapidly becoming recognized as promising treatment platforms [35].

Probiotics are living microorganisms which, when ingested, can improve or restore physiological intestinal microbial composition, thereby providing health benefits [36,37]. Probiotics are mainly Gram-positive bacteria, including various species of the genera, *Lactobacillus* and *Bifidobacterium* [38]. In particular, lactic acid bacteria (LAB) are generally distributed as natural microbiota in various fermented foods, such as kimchi, as well as dairy products, beverages, meats, wine, fruits, and plants [39,40]. LAB can have several health benefits, along with nutritional advantages, including reduction in body fat and cholesterol, prevention and control of infections, and control of certain cancers, among other conditions [41,42]. *Limosilactobacillus fermentum* is an important LAB with probiotic properties [43], and is commonly recognized as a safe bacterium, and thus used to trigger food fermentation. Further, *L. fermentum* offers technological advantages, such as enhancing the flavor and texture of food products, as well as having probiotic benefits, including anti-infection and anti-inflammatory properties [44]. In our previous study, we found that *L. fermentum* HY7302 (HY7302; 1 × 10^9^ colony forming units (CFU)/kg/day) significantly suppressed the corneal fluorescence score (CFS), as well as activating tear production and tear break time (TBUT) in Balb/c mice with BAC-induced corneal damage [45]. In addition, HY7302 increased microbiota beta diversity and altered the microbiome composition in dry eye model mice [46]; however, the underlying effects of HY7302 on ocular health remains unclear. In this study, we explored the effects of HY7302 exosomes on human conjunctiva cells. The aim of the present study was to explore whether exosomes isolated from HY7302 can improve BAC-induced inflammation in the human conjunctival cell line, clone 1-5c-4.

## 2. Results

### 2.1. Isolation of Exosomes from HY7302

**Figure 1:**
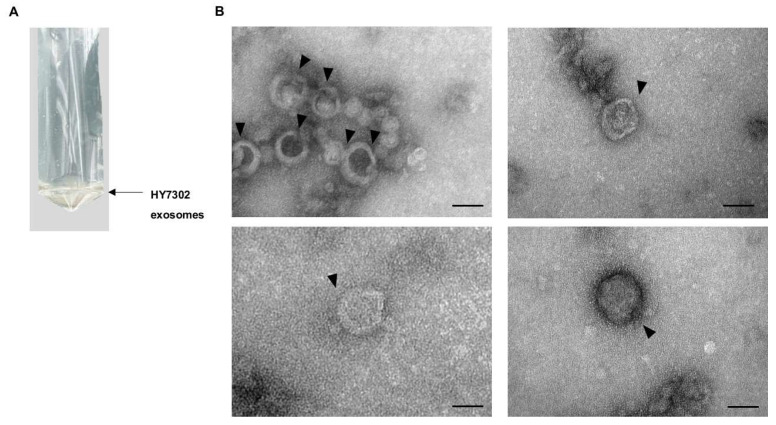
Images showing the morphology of exosomes from *Limosilactobacillus fermentum* HY7302 (HY7302). (**A**) HY7302 exosomes isolated by high-speed centrifugation. (**B**) Negative staining transmission electron microscopy images of exosomes isolated from *L. fermentum* HY7302. Scale bar, 50 nm. Arrowheads, exosomes.

In this study, the exosomes were isolated from HY7302 culture supernatant using a standard optimized high-speed centrifugation method (Figure 1A) [47], and their morphological properties were analyzed by electron microscopy. As shown in Figure 1B, HY7302 exosomes were spheroid, measured approximately 50–150 nm, and exhibited a central depression, which is a characteristic of exosomes. In addition, although the internal structure and cargo of the exosomes could not be detected, the fact that they were formed of lipid bilayers supported the inference that these particles were exosomes.

### 2.2. Physiological Properties of Exosomes Isolated from HY7302

**Figure 2:**
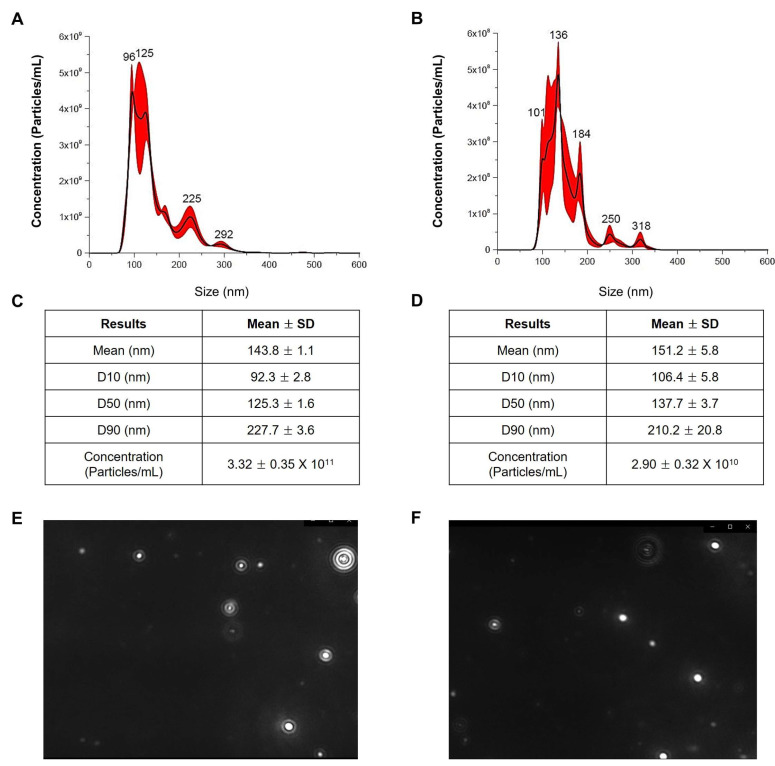
Characterization of exosomes from *Limosilactobacillus fermentum*. Nanoparticle tracking analysis of exosomes isolated from *L. fermentum* HY7302 (**A**) and KCTC3112 (**B**). Table box below show size distribution data obtained using a Malvern NanoSight NS300 and NanoSight NTA 3.4 Analytical software exosomes isolated from *L. fermentum* HY7302 (**C**) and KCTC3112 (**D**). Nanoparticle image of the isolated exosome from (**E**) HY7302 and (**F**) KCTC3112 were obtained using NTA analysis.

To investigate the size distribution of exosomes, including exosomes isolated from HY7302 and *L. fermentum* Korean Collection for Type Cultures (KCTC) strain 3112 (KCTC3112), we conducted nanoparticle tracking analysis (NTA) (Figure 2). Exosome suspension samples from HY7302 contained 3.32 ± 0.35 × 10^11^ particles/mL, with a size range of 89–231 nm, while those from KCTC contained 2.90 ± 0.32 × 10^10^ particles/mL, and ranged in size from 100 to 231 nm. Mean HY7302 exosome size was 143.8 ± 1.1 nm, while that of KCTC3112 exosomes was 151.2 ± 5.8 nm. These data suggest that exosomes isolated from the same *L. fermentum* species by high-speed centrifugation may differ in mean size, distribution, and concentration. We also quantified exosome yield using a pseudo-Lowry assay method, which is an indirect quantification technique used to infer the concentration of exosomes based on exosome membrane proteins. The protein concentration of HY7302 exosomes was 21.37 μg/μL, while that of KCTC3112 exosomes was 6.51 μg/μL suggesting that the HY7302 exosomes can effectively and stably produce exosomes at a high concentration.

### 2.3. Cytotoxicity of Exosomes Isolated from HY7302

**Figure 3:**
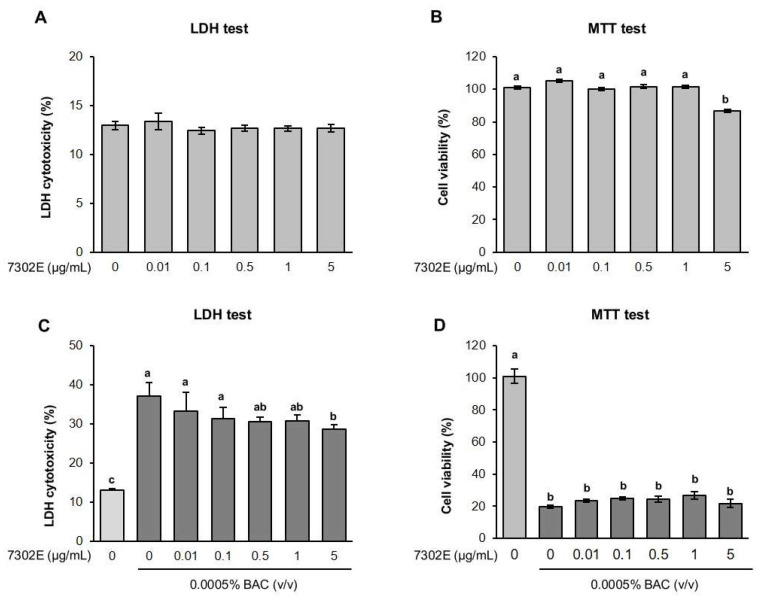
Effect of exosomes from *Limosilactobacillus fermentum* HY7302 (7302E) on cytotoxicity. (**A**,**C**) Lactate dehydrogenase (LDH) release cytotoxicity assay and (**B**,**D**) 3-(4,5-dimethylthiazol-2-yl)-2,5-diphenyltetrazolium bromide (MTT) test. Control cells or cells exposed to 0.0005% benzalkonium chloride (BAC) for 3 h were treated with *L. fermentum* HY7302 exosomes (7302E; 0, 0.01, 0.1, 0.5, 1.0, or 5.0 µg/10^4^ cells). Data are expressed as mean ± standard deviation (SD) (n = 3). Different letters indicate significantly different values (*p* < 0.05) (a > ab > b > c).

Cells from the human conjunctival line, clone 1-5c-4 (10^4^/well), were treated with 0.01–5 µg/mL of HY7302 exosomes, followed by evaluation using lactate dehydrogenase (LDH) and 3-(4,5-dimethylthiazol-2-yl)-2,5-diphenyltetrazolium bromide (MTT) assays, to test for cytotoxicity. The results of the LDH assay showed no significant cytotoxicity of HY7302 exosomes up to 5 µg/mL, whereas MTT test data showed that HY7302 exosomes had no cytotoxicity up to 1 µg/mL (Figure 3A,B). We also examined the protective effect of HY7302 exosomes against BAC cytotoxicity, which induces dry eye in human conjunctiva cells. Cells were pre-treated with HY7302 exosomes (0.01–5 µg/mL) for 24 h and then treated with 0.0005% (*v*/*v*) BAC for 3 h. LHD test showed that BAC treatment increased cytotoxicity in human conjunctival cells to 37.03%, compared with 12.96% in control cells, while HY7302 exosomes significantly reduced this cytotoxicity in a concentration-dependent manner, with a reduction of 28.55% at 1 µg/mL relative to the BAC group (Figure 3C). Further, the MTT test data showed that BAC treatment dramatically decreased conjunctival cell viability from 100% to 19.67% (Figure 3D); however, cell viability slightly increased to 26.65% when cells were treated with HY7302 exosomes at 1 µg/mL.

### 2.4. Effects of Exosomes Isolated from HY7302 on Tight Junction Molecules

**Figure 4:**
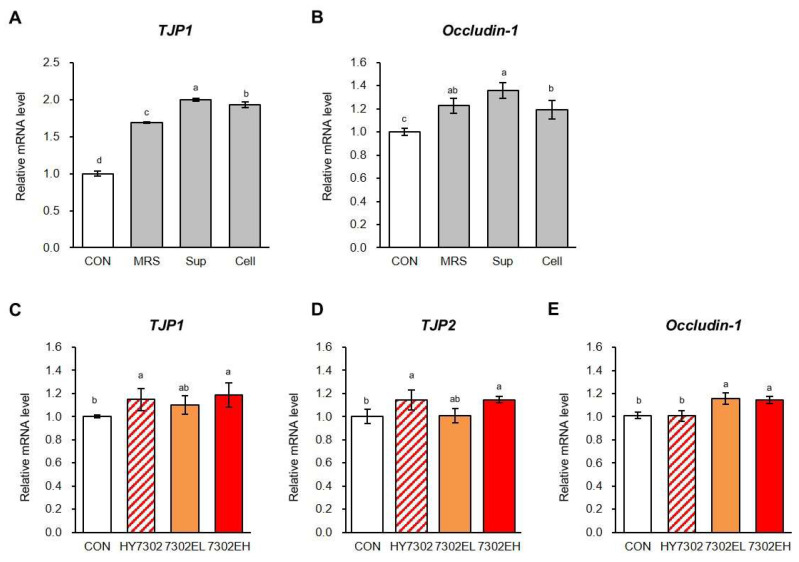
Effect of *Limosilactobacillus fermentum* HY7302 exosomes on tight junction molecules in Caco-2 cells. (**A**) Tight junction protein 1 (*TJP1)* and (**B**) occludin-1 were normalized to those of *GAPDH* and relative fold changes in their levels calculated. Levels of mRNA encoding (**C**) *TJP1*, (**D**) *TJP2*, and (**E**) occludin-1 were normalized to those of *GAPDH* and relative fold changes in their levels calculated. CON, control; HY7302, 10^6^ CFU/mL HY7302; 7302EL, 0.5 μg/mL of HY7302 exosomes; 7302EH, 1 μg/mL of HY7302 exosomes. Data are expressed as mean ± standard deviation (SD) (n = 3). Different letters indicate significantly different values (*p* < 0.05) (a > ab > b > c > d).

To assess the ability of HY7302 to modulate the gut–eye axis, we next evaluated the effect of HY7302 exosomes on regulation of intestinal tight junctions in differentiated human intestinal Caco-2 cells. Before investigating the effect of HY7302 exosomes, cells were treated with cell pellets and supernatants of 10^6^ CFU/mL HY7302 cultured in de Man Rogosa and Sharpe (MRS) broth and the effects were compared with those of MRS broth alone, as a control. As shown in Figure 4A,B, quantitative real-time polymerase chain reaction (qRT-PCR) analysis indicated that treatment with HY7302 culture supernatant increased the expression of genes (*TJP1* and *occludin-1*) encoding two tight junction proteins higher than the response to MRS medium or HY7302 cell pellet. In cells treated with HY7302 supernatant, *TJP1* and *occludin-1* mRNA levels were significantly increased by 2.00-fold and 1.35-fold, respectively, while they were upregulated by 1.92-fold and 1.19-fold in Caco-2 cells treated with HY7302 cell pellets. Subsequently, we assessed whether HY7302 exosomes had regulatory effects on epithelial intestinal cells by treating confluent CaCo-2 cells with increasing concentrations of HY7302 exosomes (0.5–1 μg/mL HY7302 probiotics as a positive control. Levels of *TJP1* and *TJP2* mRNA were 1.14-fold higher in both HY7302 groups than those in the control group, and were significantly higher (by 1.19- and 1.15-fold, respectively) in the group treated with 1 μg/mL HY7302 exosomes. In addition, HY7302 exosome-treated cells exhibited dose-dependent upregulation of the expression of both genes. By contrast, a non-significant increase (1.16-fold) in levels of *OCLN* was detected following HY7302 treatment, while its expression was significantly increased by 1.45-fold after addition of HY7302 exosomes.

### 2.5. Anti-Inflammatory Effect of Exosomes Isolated from HY7302

**Figure 5:**
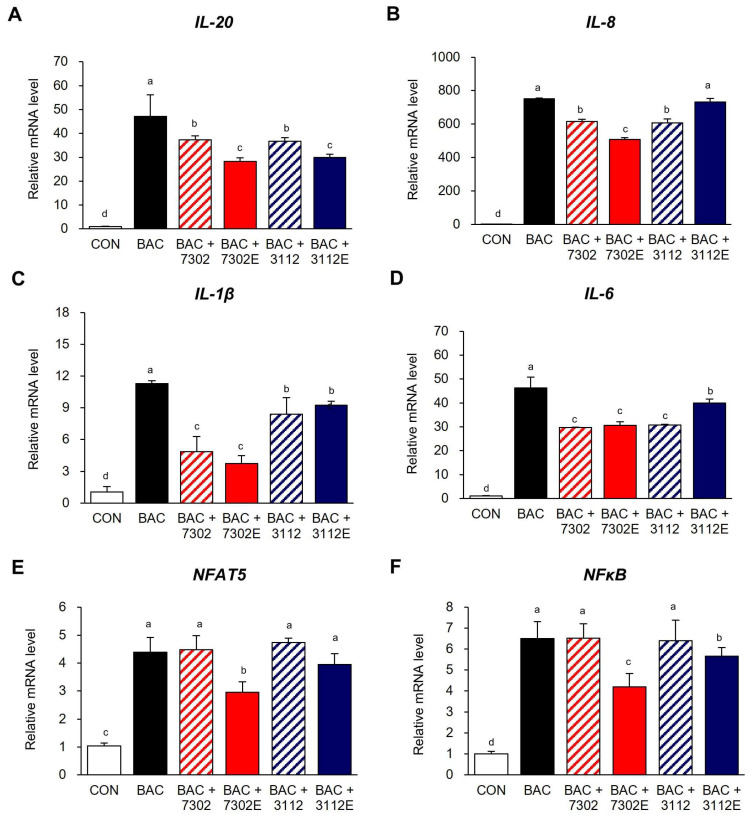
Effects of *Limosilactobacillus fermentum* HY7302 extracellular vesicles (7302E) on pro-inflammatory factors in conjunctival cell lines treated using 0.0005% BAC. Levels of (**A**) interleukin-20 (*IL20*), (**B**) *IL8*, (**C**) *IL1B*, (**D**) *IL6*, (**E**) nuclear factor of activated T cells 5 (*NFAT5*), and (**F**) nuclear factor kappa B subunit 1 (*NFKB1*) mRNA were normalized to those of *GAPDH* and calculated as relative fold-change values. CON, control; BAC, 0.0005% (*v*/*v*) BAC; 7302, 10^6^ CFU/mL HY7302; 7302E, 1 μg/mL of HY7302 exosomes; 3112, 10^6^ CFU/mL KCTC3112; 3112E, 1 μg/mL of KCTC3112 exosomes. Data are expressed as mean ± SD (n = 3). Different letters indicate significant differences (*p* < 0.05) (a > b > c > d).

In a previous study, we found that the abundance of inflammatory cytokines increased significantly after treatment of human conjunctiva cell lines with 0.0005% (*v*/*v*) of the dry eye-inducing substance, BAC [45], while treatment with 10^6^ or 10^7^ CFU/mL HY7302 probiotics could inhibit these pro-inflammatory responses in conjunctival cells treated with BAC. In this study, to determine whether the exosomes isolated from HY7302 LAB had functional effects, we analyzed the anti-inflammatory efficacy of 1 µg of exosomes isolated from HY7302 probiotics and 10^6^ CFU of HY7302 LAB. In addition, the effects of 1 µg exosomes isolated from 10^6^ CFU KCTC3112 or HY7302 were compared with those of HY7302 probiotics. As illustrated in Figure 5, BAC treatment increased the expression of pro-inflammatory cytokine genes, including *IL20*, *IL1B*, and *IL6*, by 47.1, 11.2, and 46.3-fold, respectively, while levels of the inflammatory chemokine, *IL8*, increased by > 700-fold. Treatment of HY7302 probiotics to BAC-treated cells slightly decreased *IL8*, *IL20*, *IL1B*, and *IL6* mRNA levels by 0.83-, 0.79-, 0.37-, and 0.60-fold, respectively. However, treatment with HY7302 exosomes significantly reduced *IL8*, *IL20*, *IL1B*, and *IL6* mRNA levels by 0.66-, 0.60-, 0.25-, and 0.64-fold relative to BAC-only treated cells. Meanwhile, treatment with KCTC3112 reduced levels of the corresponding cytokines by 0.78-, 0.78-, 0.66-, and 0.61-fold, which was similar to the effects of HY7302 probiotics, except for the difference in the effect on IL1B. However, the levels of IL20 (0.95-fold) in cells treated with KCTC3112 exosomes and BAC did not differ significantly from those of cells treated with HY7302 exosomes. Meanwhile, KCTC3112 exosomes showed a significant difference from HY7302 exosomes, as there was no significant difference from the BAC treatment group for IL8 and IL6. Levels of nuclear factor of activated T cells 5 (*NFAT5*) and nuclear factor kappa-light-chain-enhancer of activated B cells (*NFKB1*) mRNAs, which encode proteins that stimulate the expression of various pro-inflammatory cytokines, were also investigated. *NFAT5* and *NFKB1* levels were significantly higher following BAC treatment, rising by 4.24- and 6.47-fold; meanwhile, among the treatments tested, only HY7302 exosomes significantly reduced their levels by 0.67- and 0.64-fold.

### 2.6. Exosomes Isolated from HY7302 Are Taken Up by Conjunctival Cells in a Transwell System

**Figure 6:**
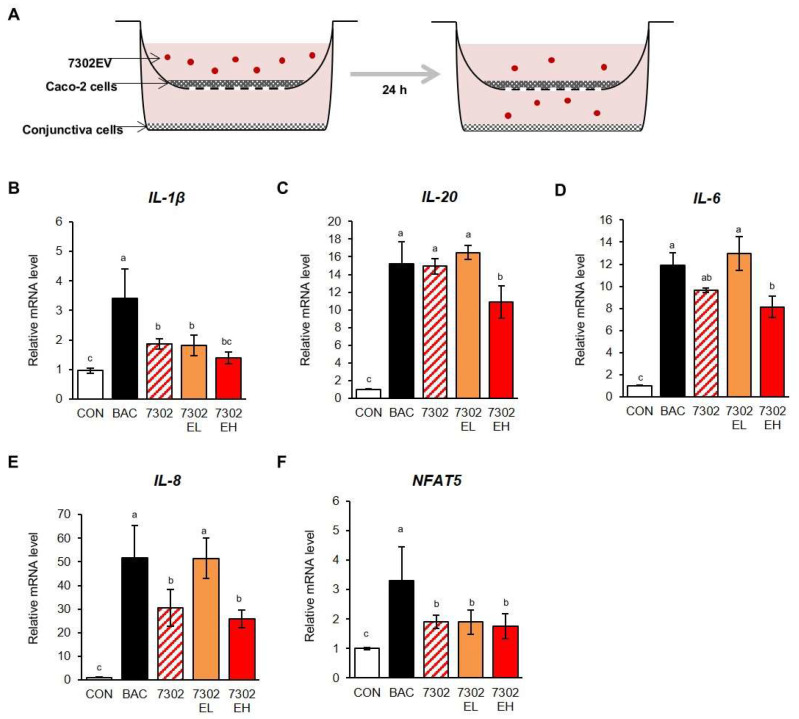
(**A**) Schematic of the experimental protocol to test the effect of exosomes of *Limosilactobacillus fermentum* HY7302 (7302E) on pro-inflammatory cytokines production. Clone 1-5c-4 cells were seeded in transwell plates and, once they reached confluence, exposed to Caco-2 cells. Caco-2 cells were co-cultured either with or without exosomes, using transwells, and culture medium samples of conjunctiva cell collected for analysis. Levels of (**B**) *IL1B*, (**C**) *IL20*, (**D**) *IL6*, (**E**) *IL8*, and (**F**) *NFAT5* mRNA in cells were normalized to those of *GAPDH* and relative fold-change values calculated. CON, control; BAC, 0.0005% (*v*/*v*) BAC; 7302, 10^6^ CFU/mL HY7302; 7302E, 1 μg/mL of HY7302 exosomes; 3112, 10^6^ CFU/mL KCTC3112; 3112E, 1 μg/mL of KCTC3112 exosomes. Data are expressed as mean ± SD (n = 3). Different letters indicate significant differences (*p* < 0.05) (a > ab > b > bc > c).

Next, analysis was performed using transwell plates consisting of two culture chambers separated by porous membrane filters. We investigated whether exosomes isolated from HY7302 LAB acted as regulators of cell-to-cell communication. To determine whether the exosomes added to Caco-2 intestinal cells could migrate and affect the conjunctival cells, we used membrane filters with 0.4 μm pores. As shown in Figure 6A, exosomes were added to Caco-2 cell culture medium, and conjunctival cells were treated by incubation with 0.0005% BAC for the last 3 h of the experiment. Levels of mRNAs encoding the inflammatory cytokines, IL-1b, IL-20, IL-6, and *IL-8* were significantly higher (3.42-, 15.17-, 11.62-fold, and 51.66-fold, respectively) in the BAC-treated group than those in the control group. Low concentrations of HY7302 exosomes (0.5 µg/mL) only significantly reduced levels of *IL1B* mRNA (1.81-fold), but did not alter those of *IL6*, *IL20*, and *IL6*; however, levels of *IL1B*, *IL20*, *IL6*, and *IL8* mRNA were significantly lower following treatment with a high concentration of HY7302 exosomes (1 µg/mL) than those in BAC-treated cells (1.38-, 10.88-, 8.36-, and 25.79-fold, respectively). Treatment with 10^6^ CFU/mL HY7302 probiotics as a control also led to significantly lower expression levels of *IL1B*, *IL6*, and *IL8* (1.86-, 9.76-, 30.54-fold, respectively), but not those of *IL20*. Finally, levels of the gene encoding NFAT5 in cells treated with HY7302 or both concentrations of HY7302 exosomes (0.5 and 1 µg/mL) were significantly lower than those in the BAC group, but did not differ significantly from one another. In summary, our data show that 1 µg/mL HY7302 exosomes could significantly attenuate BAC-induced pro-inflammatory cytokine expression in conjunctival cells, and were more effective than the HY7302 probiotic.

## 3. Discussion

Exosomes are biological extracellular EV that are naturally secreted by mammalian and plant cells [48], with diameters of 30–160 nm and densities of 1.1–1.2 g/mL [49]. Exosomes comprise a lipid bilayer structure enclosing small molecule components, such as proteins, nucleic acids, lipids, and secondary metabolites [50,51]. The release of these intracellular cargoes can induce physiological, phenotypic, and functional changes in recipient cells [28]. Interestingly, exosomes were previously considered to be a means of transport for removal of waste from cells [52]; however, it has since been established that they act as regulators of cell-to-cell communication through gene expression regulation, as well as by delivering biologically active substances [53].

Numerous studies have focused on exosomes as novel biomarkers or potential therapeutics [54,55]. For example, plant-derived exosomes can have protective effects in immunity against pathogen invasion [56]. In addition, microbial-derived exosomes can also act as natural antipathogens or food preservatives, as well as having anti-inflammation, anti-obesity, anti-alcoholic-related liver disease, and anti-gut barrier dysfunction effects [31,57,58]. *Bifidobacterium longum* NCC2705 releases numerous nanoparticles, made up of lipid bilayers, into the extracellular environment [59]. Further, *B. longum* AO44 secretes exosomes, which can promote intestinal bacterial immunomodulatory and anti-inflammatory effects on the host through IL-10 and IL-17 [60]. Additionally, exosomes from *Lactiplantibacillus plantarum* regulate skin aging by inhibiting wrinkle formation and pigmentation, and reduce mRNA expression levels of *MMP1*, an inflammatory factor [32]. Hence, various gut microbes have the potential to impact health in numerous ways, among which important mechanisms related to probiotic-induced therapeutic effects include communication to influence inflammation and the immune system [36,61]; however, the immunomodulatory molecules involved in this mode of symbiotic LAB–host communication remain largely unknown.

DED, or keratoconjunctivitis sicca, is a multifactorial ocular surface disease, characterized by decreased tear production [3]. Ocular inflammation is a critical factor in DED, which is accompanied by eye irritation, hyperemia, glare, eye fatigue, and blurred vision [2,62]. Inflammation of the ocular epithelial cells induces these complications through a cascade of increased cytokines [63]. Traditionally, lubricating eye drops and ointments have been used to treat dry eye, as well as topical anti-inflammatory therapies; however, common ocular anti-inflammatory drugs, such as corticosteroid eye drops or cyclosporine, have various side effects in patients with dry eye, making them generally difficult to use [64,65]. Therefore, research on functional health foods, with the aim of identifying novel, safe, and effective anti-inflammatory therapies for DED, has attracted attention. In this study, we aimed to provide a basis for a therapeutic approach in which HY7302-derived exosomes can regulate the production of inflammatory cytokines in the pathogenesis of inflammatory eye disease. In our previous paper, we observed that HY7302 intake could suppress corneal damage and improve the function-related tear production in BAC-induced dry eye model mice. Further, the HY732 probiotic altered the microbiota composition by influencing beta diversity and increasing the abundance of *Bifidobacterium pseudolongum* [46]; however, there are no previous reports on which functional molecules of *L. fermentum* affect eye health. Therefore, we investigated whether HY7302 exosomes could exert specific functional effects that alter the inflammatory response of conjunctival cells by acting on the gut–ocular axis. In particular, the conjunctival tissue is rich in blood vessels, lymphatic vessels, and nerve fibers [66]. In addition, conjunctival cells are closely related to tear secretion because the secretions from the lacrimal glands flow into the conjunctival sac, and are drained by the lacrimal ducts of the upper and lower eyelids [67]. Therefore, we suggest that the exosomal functional components of probiotics can circulate to the conjunctival tissue, which may ultimately act directly to improve dry eye and corneal damage.

It is established in the literature that it is important to isolate exosomes of high purity and with high yield in a state close to that occurring in vivo. There are various methods for isolating and purifying exosomes, such as high-speed centrifugation and polymer precipitation [68,69]. In this study, we used high-speed centrifugation to isolate high-purity exosomes from HY7302 culture supernatant, which is the most commonly used method for isolating exosomes by size. Moreover, we isolated exosomes from both HY7302 and KCTC3112 in the same way and compared their physiological properties. Data generated by NTA showed that the number of extracellular particles isolated from HY7302 differed from that isolated from KCTC3112 visible in the same optical area. We concluded that smaller exosomes were actively produced by HY7302, as the average sizes of particles extracted from HY7302 in each size range were smaller than those from KCTC3112, except among the top 10% of total exosome particle size. In addition, HY7302-derived exosomes were observed via transmission electron microscopy (TEM), which is a basic method used to characterize particle size. The diameters of the HY7302 particles inferred to be exosomes, based on their double-membrane structure observed in TEM images, were approximately 50–150 nm.

Tight junctions are cell–cell adhesion complexes that are strongly developed in intestinal tissues and form intercellular barriers [70]. When tight junctions leak, due to external invasion or imbalance of the intestinal microbiome, inflammatory cytokines can escape into other tissues and induce immune and inflammatory responses [71]. The tight junction proteins, TJP1 and TJP2, are cytoplasmic peripheral membrane proteins with multiple domains specialized for protein interaction that are required for assembly of both adherences and tight junctions [72]. Occludin-1 is also an essential tight junction component, which seals the paracellular space and prevents unrestricted leakage [73]. To confirm that exosomes play an important role in the effects of HY7302 on eye health via the gut–eye axis, we evaluated their effects on the regulation of tight junctions in human intestinal Caco-2 cells. Our data demonstrate a potentially important effect of HY7302 supernatant in enhancing tight junction barrier gene levels in the cells. Indeed, we established that the anti-inflammatory bioactive effect of culture supernatant was stronger than that of the bacterial cells themselves. Relative to the controls treated only with HY7302, cell pellets, or MRS, culture supernatant substantially increased levels of *TJP1* and *OCLN*. We inferred from these findings that the active substance derived from HY7302 is present in the culture supernatant or bacterial cells, and therefore investigated the effect of isolated HY7302 exosomes in the supernatant in Caco-2 cells on gut junction molecules. Both low (0.5 µg/mL) and high (1 µg/mL) concentrations of HY7302 exosomes were used to treat confluent Caco-2 cells for 24 h; HY7302 probiotics served as a positive control. Levels of mRNAs encoding TJP1, TJP2, and occludin-1 were significantly higher after treatment with 0.5 and 1 μg/mL HY7302 exosomes than those in untreated or probiotic-treated cells. Based on the above results, it is most likely that HY7302-derived exosomes enhance the integrity of intercellular junctions by regulating the expression of junction molecules, leading to enhanced tight junction interactions between endothelial cells.

As understanding of the role of inflammation in DED increases, improvement in the changes to the ocular surface through the consumption of functional foods is becoming a focus of attention as an alternative to use of tear replacements [74]. Ocular epithelial cells exposed to certain concentrations of BAC exhibit increased inflammatory cytokine production [11]. In addition, BAC-induced DED has been reported to be accompanied by increased production of inflammatory chemokines in several studies. For example, increases in the concentrations of inflammatory cytokines, such as IL-1B, Il-6, IL-8, and tumor necrosis factor-α (TNF-α), in tears are correlated with clinical indices of dry eye [19]. In addition, IL-20 was significantly increased in the tears of animal models with BAC-induced dry eye; as IL-20 can induce an inflammatory response, including infiltration and activation of macrophages, it has been suggested that it is a potential treatment target in patients with dry eyes [75]. Moreover, the expression of these pro-inflammatory cytokines can be regulated by NF-κB and NFAT5. NF-κB is a member of a family of inducible transcription factors that regulate numerous genes involved in various immune and inflammatory response processes [63]. In addition, NFAT5 is an important regulator of the expression of NF-κB, which is a key inflammatory response modulator [76]. In this study, we found that the mRNA levels of IL-20, IL-8, and IL-1B of the cell treated with 1 µg/mL HY7302 exosomes significantly lower than those of the cell treated with 10^6^ CFU/mL HY7302 probiotics. In this study, we found that treatment of cells induced with inflammation by BAC with 1 µg/mL HY7302 exosomes significantly reduced the mRNA levels of IL-20, IL-8, and IL-1B as much as when treated with 10^6^ CFU/mL HY7302 probiotics. In addition, *NFAT5* and *NFKB1* mRNA expression levels were strongly induced by BAC treatment, but dramatically decreased in cells treated with HY7302 exosomes, with levels much lower than those in cells treated with KCTC3112 exosomes. Together, our data show that HY7302 exosomes have the potential to prevent BAC-induced multifactorial ocular dysfunction by reducing inflammatory cytokine expression. We also investigated the anti-inflammatory responses of intestinal and ocular conjunctival cells to HY7302 exosomes, by the coculture of both types of cell line in a transwell system. Treatment of Caco-2 cells with 0.5 and 1 µg/mL HY7302 exosomes significantly reduced transcription of all the intercellular cytokines tested, with levels of *IL20*, *IL6*, and *IL8* markedly lowered. These results indicate that exosomes derived from HY7302 probiotic can migrate to ocular epithelial cells and suppress BAC-related inflammation. Furthermore, our findings confirm that these exosomes can act as anti-inflammatory regulators between gut and ocular tissues, suggesting that exosomes are functional agents of *L. fermentum* HY 7302.

The number of patients with dry eye has increased dramatically over the past few decades. Dry eye disease can cause inflammatory responses in ocular and conjunctiva tissues. In our previous study, we analyzed changes in the intestinal microbiome and changes in inflammatory markers in intestinal tissue, among several factors that may affect distant organs when orally consumed probiotics. In this study, we demonstrated that the exosomes of HY7302 can pass through differentiated enterocytes and directly reach and affect conjunctival cells. This provides clues to how HY7302 acts (MOA) to directly improve dry eye syndrome in addition to regulating the microbiome and intestinal immunity. Further investigations to clarify the physiological characteristics of the extracellular particles involved, by revealing which proteins, DNA, RNA, or organic acids, etc., are present in exosomes from HY7302 probiotics, are warranted. These extracellular secreted exosomes are important factors in host–microbe interactions; however, it will also be necessary to determine whether they act as specific factors that enable HY7302 attachment and survival in the gastrointestinal tract. Our results highlight the importance of HY7302 exosomes and their potential for development as a future therapeutic approach for dry eye and conjunctival inflammation. Furthermore, the data suggest that certain exosomes isolated from HY7302, which have been proven to be safe, may be utilized as effective materials of potential value as treatments for dry eyes or functional health foods. It is easily applicable in clinical practice and would help establish new clinical trials for dry eye using supplementation with HY7302 probiotics

## 4. Materials and Methods

### 4.1. HY7302 Cell Culture and Exosome Isolation

*L. fermentum* HY7302 was isolated from raw milk obtained from Korean farms. *L. fermentum* type strain KCTC 3112 was obtained from KCTC. Both *L. fermentum* strains were cultured in MRS medium (BD Difco, Detroit, MI, USA; KisanBio, Seoul, Republic of Korea) at 37 °C for 18 h. For in vitro studies, *L. fermentum* cultures were centrifuged (2000× *g*, 20 min), and then culture supernatant medium was centrifuged again (2000× *g*, 4 °C, 20 min). After centrifugation, cell pellets were washed twice with PBS, and then resuspended in PBS. Culture supernatant and cell pellet samples were prepared to a final concentration of 1  ×  10^6^ CFU/mL and used for in vitro experiments.

Exosomes were isolated from culture supernatants by sequential high-speed centrifugation using a total exosome isolation commercial kit (Cat no. 4478359, Thermo Fisher Scientific, Waltham, MA, USA). Briefly, HY7302 and KCTC3112 cell supernatant samples were centrifuged (2000× *g*, 20 min, 4 °C) and cell debris removed. Supernatants were transferred to new tubes and then mixed with kit reagents at a 1:2 ratio before incubation overnight at 4 °C. Exosome pellets were washed with PBS and subjected to high-speed centrifugation (10,000× *g*, 60 min, 4 °C). HY7302 and KCTC3112 exosomes samples were resuspended in PBS, aliquoted, and stored at −80 °C until further use.

### 4.2. Detection of Total Protein Content in HY7302 Exosomes

Protein concentrations in exosomes were measured using a DC Protein Analysis Kit (BIO-RAD, Hercules, CA, USA), according to the manufacturer’s instructions; absorbance was measured at 750 nm on an ELISA microreader (BioTek, Winooski, VT, USA). Based on the results of this analysis, 50 µg/mL stock solutions of HY7302- and KCTC3112-derived exosomes were prepared in PBS, and added to cells at final concentrations of 0.5 or 1 μg/mL for 24 h.

### 4.3. Cell Culture

The human conjunctival epithelial cell line, clone 1-5c-4, was seeded at  1 ×  10^5^ cells/mL and cultured in Dulbecco’s modified Eagle’s medium (DMEM)/F-12 containing 10% *v/v* fetal bovine serum (FBS; Gibco, Thermo Fisher Scientific, Waltham, MA, USA) and 1% antibiotic/antimycotic in a 5% CO_2_ humidified air atmosphere at 37 °C. For treatments, 1  ×  10^9^ cells/mL stock solutions of HY7302 or KCTC3112 were prepared in PBS, and added to cells at a final concentration of 1  ×  10^6^ CFU/mL. HY7302 and KCTC3112 exosomes were prepared in PBS and added to cells at final concentrations of 0.5 and 1 μg/mL for 24 h. To generate a dry eye model, clone 1-5c-4 cells were treated with 0.0005% (*v*/*v*) BAC for the last 3 h.

Caco-2 cells were also seeded at 1 × 10^5^ cells/mL and maintained in DMEM containing 10% *v/v* FBS and 1% antibiotic/antimycotic in a 5% CO_2_ humidified air atmosphere at 37 °C. Cells were cultured and fully differentiated for 21 days. Caco-2 cells were treated with culture supernatants and cell pellets of HY7302 and KCTC3112 probiotics at 1  ×  10^6^ CFU/mL for 24 h.

### 4.4. Cell Viability Protection and Toxicity Assays

The effects of HY7302 exosomes on cell viability were determined using MTT and LDH assays. Conjunctival epithelial cells were seeded in 96-well plates at 1  ×  10^4^ cells/mL and incubated in serum-free medium, then treated with various concentrations of HY7302 exosomes (0.01, 0.1, 1, 0.5, and 5 μg/mL) for 24 h. For the MTT test, 0.5 mg/mL MTT stock solution was added and incubated for 4 h. After MTT-containing medium was aspirated, 200 μL of DMSO was added for elution of formazan crystals. Then, absorbance values at 590 nm were measured using a microreader (BioTek, Winooski, VT, USA). Cytoplasmic LDH activity was detected in cell-free supernatant samples from HY7302 exosome-treated cells using an LDH assay kit (G1780; Promega, Madison, WI, USA), according to the manufacturer’ instructions. LDH assay results are expressed as percentage cell viability relative to that of positive control-treated controls, which was set at 100%.

### 4.5. NTA of HY7302 Exosomes

The exosome size distributions and concentrations were detected using a Nanosight NS300 instrument (Malvern Panalytical Ltd., Malvern, UK, version no. 3.4). Fractions were diluted 1:100 or 1:1000 with sterile PBS, to ensure that the number of detectable particles was within the optimal range of 106–109 particles/mL. NanoSight NTA 3.4 analytical software was used to accurately quantify the size and concentration of exosomes in samples; each sample was analyzed in triplicate.

### 4.6. TEM Analysis of HY7302 Exosomes

Exosomes freshly isolated from HY7302 culture supernatants were resuspended in Dulbecco’s PBS. HY7302 exosomes were fixed using 4% (*v*/*v*) paraformaldehyde, then dropped onto a copper grid and stored at room temperature for 1 min before removal of the liquid using filter paper. Samples were then treated with uranyl acetate for 1 min and then remaining liquid was removed with filter paper. Prepared samples were then dried at room temperature for further observation. TEM was conducted using a JEM-1400 (JEOL Co., Ltd., Tokyo, Japan). The exosome sizes were determined using RADIUS software (EMSIS, Version 3.0.26).

### 4.7. Transwell Coculture of Caco-2 and Clone 1-5c-4 Cells

Briefly, fully differentiated Caco-2 cells (1 × 10^5^ cells/well) and 0.5 or 1.0 μg/mL HY7302 exosomes were seeded in the upper chambers of 6-well 24 mm transwell plates with 0.4 μm pore filters (Costar, Washington, DC, USA). Conjunctival clone 1-5c-4 cells without exosomes were added to the lower chambers. After incubation for 24 h, cells in the lower chamber were exposed to 0.0005% (*v*/*v*) BAC for 3 h. Aliquots of medium and harvested cells were collected from the lower chamber and stored at −80 °C until further use. Each experiment was repeated three times.

### 4.8. qRT-PCR

RNA was extracted using Trizol reagent (iNtRON Biotechnology, Seongnam-si, Republic of Korea) according to the manufacturer’s instructions, and cDNA synthesized from 600 ng total RNA using a commercial kit (Maxime RT PreMix Kit; iNtRON Biotechnology). Then, cDNA was analyzed by qRT-PCR using the TaqMan Probe-Based Gene expression assay system (Applied Biosystems, Carlsbad, CA, USA) and TaqMan Gene Expression Master Mix (Applied Biosystems, Waltham, MA, USA). Quantification of *TJP1* (Hs01551871_m1), *TJP2* (Hs00910543_m1), occludin-1 (Hs05465837_g1), *IL20* (Hs00218888_m1), *IL1B* (Hs01555410_m1), *IL8* (Hs00174103_m1), *IL6* (Hs00174131_m1), *NFAT5* (Hs00232437_m1), and *NFKB1* (Hs00765730_m1) transcripts was performed using gene-specific primers. All mRNA data were normalized to *GAPDH* (Hs99999905_m1) levels. To compare levels between samples, relative mRNA levels were calculated using the 2^(−ΔΔCT)^ method.

### 4.9. Statistical Analysis

At least three independent NTA analyses were performed, and data on particle amounts and diameters are presented as mean ± standard deviation (SD) values. Cell toxicity, viability data, qRT-PCR and ELISA data were analyzed using one-way ANOVA and Duncan’s test (SPSS, version 18.0, Chicago, IL, USA) and are presented as mean ± SD. *p* < 0.05 was considered significant.

## 5. Conclusions

Although gut–eye axis communication provides an attractive perspective for interpretation of mechanisms occurring in health and disease, the role of L. fermentum in this context has remained relatively unexplored. In the present study, we identified the properties and activities of functional substances from HY7302 probiotics, which have previously been shown to improve dry eyes using in vivo experiments. We observed that HY7302 produced exosomes were of a smaller mean particle size and present in larger amounts than those from a standard strain of the same species, KCTC3112. In addition, we provide evidence that HY7302-derived exosomes both enhance tight junction barriers in intestinal cells, and reduce the expression levels of inflammatory cytokines in ocular epithelial cells. Overall, our data suggest that HY7302 probiotics could significantly improve gut–eye axis communication through exosome-mediated anti-inflammatory regulation.

## Data Availability

Data are contained within the article.
